# Global research landscape of telomere biology in infectious diseases: mechanistic links between host–pathogen interactions and immune ageing

**DOI:** 10.3389/fragi.2025.1729868

**Published:** 2026-01-08

**Authors:** Theophilus Nang Wakai, Nina Ghislaine Yensii, Fabrice Banadzem Kernyuy, Mercy Bella-Omunagbe, Shalom Nwodo Chinedu, Israel Sunmola Afolabi

**Affiliations:** 1 Department of Biochemistry, College of Science and Technology (CST), Covenant University, Ota, Nigeria; 2 Covenant Applied Informatics and Communication, Africa Centre of Excellence (CApIC-ACE), Ota, Nigeria

**Keywords:** bibliometric analysis, immune ageing, infection-related stress, infectious diseases, telomeres

## Abstract

Telomeres, nucleoprotein structures located at the ends of chromosomes, maintain genomic stability and regulate cellular lifespan, particularly in immune cells. Telomere shortening, driven by cell division and limited telomerase activity, accelerates immune ageing and increases susceptibility to infectious diseases. Chronic infections like HIV and tuberculosis exacerbate telomere attrition through sustained immune activation and oxidative stress. This study presents a bibliometric review of research on telomere length and infectious diseases from 2005 to 2025. Data from the Web of Science Core Collection were analysed using VOSviewer and CiteSpace, software tools for visualising co-authorship, citation, and keyword networks, to assess publication trends, collaborations, and themes. A total of 123 publications were identified, showing steady growth with a 60% increase in publications from 2020 to 2022 during the COVID-19 pandemic. Leading journals included Frontiers in Immunology, PLoS ONE, and Scientific Reports. The United States produced the largest share of publications, followed by Canada and Spain, with notable contributions from the University of British Columbia and Université de Montréal. Influential authors such as Côté HCF, Pick N, and Maan EJ have advanced research, particularly in the areas of HIV and tuberculosis. Keyword analysis highlighted two dominant themes: immune ageing and infection-related stress. Malaria research was comparatively scarce, underscoring a gap for future investigation. These findings inform future research on telomere-targeted interventions and epidemiological studies aimed at enhancing infectious disease management. This review provides a comprehensive overview of the field’s progress and identifies key areas for future investigation.

## 1 Introduction

### 1.1 Background on telomeres and their function

Telomeres, the protective caps at the ends of chromosomes, are crucial in maintaining genomic stability and cellular longevity. These specialised DNA-protein structures prevent chromosome degradation, fusion, and recognition as damaged DNA, thereby safeguarding the integrity of the genetic material (Lee et al., 2025). Telomeres consist of repetitive nucleotide sequences, typically TTAGGG in vertebrates, which are bound by a complex of proteins known as shelterin. This complex shields the telomeric DNA from DNA repair mechanisms and regulates telomere length (Mir et al., 2020). The shelterin complex also facilitates the formation of a protective loop structure (t-loop), further stabilising the telomere (Dufourd et al., 2025). Without telomeres, chromosomes would be susceptible to damage and degradation, resulting in genomic instability and cellular dysfunction. Telomere length (TL) is dynamic and progressively shortens with each successive cell division due to the end-replication problem (Bonnell et al., 2021). While telomerase, a specialised reverse transcriptase, replenishes telomeric DNA in germline cells, stem cells, and some immune cells, its activity is limited in most somatic cells (Lee et al., 2025). Consequently, telomere shortening serves as a biological clock of cellular ageing. Critically short telomeres activate DNA damage pathways, inducing replicative senescence or apoptosis (Eller et al., 2002; Koliada et al., 2015; Zahid, 2023). These processes contribute to tissue dysfunction and age-related diseases, including cardiovascular, metabolic, and neurodegenerative disorders. In the immune system, TL influences the replicative lifespan of lymphocytes, shaping adaptive immunity and susceptibility to disease (Chebly et al., 2023; Heba et al., 2021).

Telomere shortening, driven by cell division and limited telomerase activity, accelerates immune ageing and increases susceptibility to infectious diseases. (Chebly et al., 2023; Nassour et al., 2024). Chronic infections like HIV and tuberculosis exacerbate telomere attrition through sustained immune activation and oxidative stress (Adelakun et al., 2022). Immunosenescence, the age-related decline in immune function, is influenced by telomere shortening (Heba et al., 2021).

Rising evidence supports an interplay between infectious diseases and telomere dynamics (Ilmonen et al., 2008). Acute infections trigger robust immune activation, necessitating rapid clonal expansion of T and B cells, thereby accelerating telomere erosion (Chebly et al., 2023; Fali et al., 2019). Malaria infection remains the top global health challenge, affecting millions of people especially in subsaha Africa (Venkatesan, 2025; Olasehinde et al., 2019). Similar associations are evident in malaria, where infection-induced inflammation may accelerate telomere shortening, yet human studies remain limited compared to animal models ((Asghar et al., 2017; Miglar, 2023; Miglar et al., 2021; Wakai et al., 2025a)). Chronic infections such as HIV, hepatitis, or tuberculosis sustain prolonged immune activation, compounding telomere attrition and leading to premature immune senescence (Adelakun et al., 2022; Macamo et al., 2024; Wang et al., 2019). Oxidative stress and systemic inflammation, hallmarks of chronic infection, further exacerbate telomere erosion (Correia-Melo et al., 2014). Similar associations are evident in malaria (Miglar et al., 2021; Miglar et al., 2020), Bacterial (Noppert et al., 2020) and other viral infections such as COVID-19 (Aviv, 2020). Thus, telomere biology provides mechanistic insights into immune exhaustion and pathogen persistence.

Despite growing evidence connecting telomere dynamics with infectious diseases, the evolution of knowledge in this area has not yet been systematically mapped (Tunnicliffe et al., 2024). Bibliometric analysis provides a valuable tool for evaluating the development of research fields by quantifying publication output, collaboration networks, and citation patterns, thereby identifying global trends and gaps. In health sciences, this approach enables assessment of scientific influence, research performance, and thematic directions across countries and institutions (Tomaszewski, 2023). Applied here, it can uncover how telomere biology has been investigated in relation to infectious diseases, which pathogens and mechanisms have drawn the most attention, and where critical, underexplored areas remain (Afolabi et al., 2021). A bibliometric review published in 2020 (Valera-Gran et al., 2020) examined research on telomere length in children, and a systematic review has explored the relationship between infection and telomere length (Tunnicliffe et al., 2024). However, no study has comprehensively assessed telomere length in the context of infectious diseases. As a result, the scope, growth, and impact of research in this area remain unclear. This study analyses global research output on telomere biology and infectious diseases, maps research trends, and highlights thematic areas that can inform future scientific inquiry. By integrating bibliometric mapping with mechanistic interpretation, this review elucidates how telomere biology regulates immune homeostasis during infection and influences pathogen persistence within host–pathogen interactions.

### 1.2 Scope and objectives of the review

This bibliometric review synthesises 2 decades of research linking telomere biology with infectious diseases. The 2005–2025 period was chosen to capture the emergence of pivotal studies linking telomere dynamics to infectious diseases, as well as advancements in bibliometric tools such as VOSviewer, which enable robust analysis of research trends (Pranckutė, 2021). The objectives are:

1. To quantify publication trends and assess the growth trajectory of the field.
2. To identify leading journals, authors, institutions, and countries.
3. To highlight influential publications and citation dynamics.
4. To map thematic clusters through keyword co-occurrence, emphasising immune response and oxidative stress.
5. To contextualise findings within biological and clinical frameworks, offering directions for future research.

This review focuses on human-relevant studies, excluding animal models unless they provide direct translational insights into human telomere dynamics (e.g., Asghar et al., 2015).

## 2 Materials and methods

### 2.1 Search strategy and inclusion criteria

We searched the Web of Science (WoS) Core Collection for publications between 1 January 2005, and 20 July 2025. Search terms combined telomere biology and infectious disease constructs:

TS = (“telomere length” OR “telomere dynamics” OR “telomerase activity” OR “telomere shortening” OR “telomere attrition”)

AND TS = (“infectious disease*” OR “viral infection*” OR “bacterial infection*” OR “parasitic infection*” OR malaria OR HIV OR tuberculosis OR COVID-19 OR “chronic infection*” OR “acute infection*”)

AND TS = (“immune response” OR inflammation OR “oxidative stress” OR “leukocyte telomere length” OR “host-pathogen interaction”).

Only peer-reviewed articles and reviews in English with full bibliographic metadata were included.

### 2.2 Data source justification

This bibliometric review was conducted using the Advanced Search tool in the Web of Science (WoS) (https://www.webofscience.com/wos/woscc/advanced-search) Core Collection as the sole data source.

WoS was selected because it offers comprehensive coverage of high-impact journals, robust citation indexing, and consistent metadata formatting that facilitates advanced bibliometric analyses (Pranckutė, 2021; Vijayan DrSS, 2021; Web of Science, 2024). In addition, WoS provides backward and forward citation tracking with high reliability, which is essential for co-citation, co-authorship, and keyword network mapping (Yan and Zhiping, 2023). Although other databases, such as Scopus, PubMed, and Dimensions, index additional journals, they often present challenges, including inconsistent metadata, limited historical coverage, and increased risk of duplication when datasets are merged (Guerrero-Bote et al., 2021). Prior bibliometric studies have established WoS as the gold standard due to its compatibility with widely used software tools such as VOSviewer and CiteSpace (Tomaszewski, 2023; Web of Science, 2024). Therefore, restricting the dataset to WoS ensures both methodological rigour and comparability with similar bibliometric reviews (Klarin, 2024).

### 2.3 Exclusion criteria

The following criteria were applied to exclude studies that did not align with the research focus on the relationship between telomere dynamics and infectious diseases in human-relevant contexts:

1. Studies unrelated to infectious diseases
2. Non-human models were excluded unless they provided direct translational insights into human telomere dynamics to maintain focus on clinical relevance.
3. Non-human models without clinical translation.
4. Records lacking telomere-infection data.
5. Editorials, commentaries, and conference abstracts.
6. Duplicates or incomplete metadata.

### 2.4 Data extraction and analysis

From Web of Science, we extracted metadata including publication year, authorship, journal, institutional affiliation, country, keywords, and citations, as this database provides standardised and comprehensive metadata for bibliometric studies (Klarin, 2024). The analyses utilised:

1. VOSviewer for co-authorship, co-citation, and keyword clustering, enabling visualisation of scientific networks and thematic relationships (van Eck and Waltman, 2010).
2. CiteSpace for trend detection and thematic mapping, facilitating the identification of temporal patterns and intellectual structures in the literature (Chen, 2006).
3. Microsoft Excel for descriptive statistics, offering a robust platform for summarising bibliometric data trends (Meyer and Avery, 2009).

VOSviewer was configured with a minimum of five co-occurrences for keywords and 10 citations for co-citation analysis, using fractional counting for clustering. CiteSpace employed a time-slicing approach (2005–2025) with a minimum burst strength of 2 for trend detection (van Eck and Waltman, 2010; Chen, 2006).

Quality control measures included manual screening to remove duplicates, verification of metadata accuracy (e.g., author affiliations, publication years) via Web of Science records, and exclusion of retracted articles to ensure data integrity (Guerrero-Bote et al., 2021).

## 3 Results

### 3.1 Publication analysis based on numbers

**FIGURE 1:**
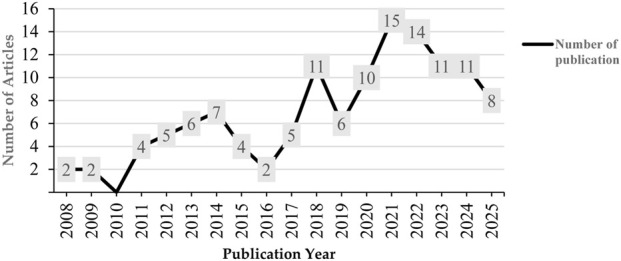
Annual scientific publication trends on telomere length dynamics and infectious diseases from 2005 to 2025. The results show a steady upward trajectory with peaks during 2020–2022, coinciding with the COVID-19 pandemic.

From 1 January 2005, to 20 July 2025, a total of 123 publications related to telomere length dynamics and infectious diseases were identified. Figure 1 depicts the annual growth of these publications, highlighting a gradual increase in a in scholarly interest has emerged in recent years. The refined dataset of 123 publications, after applying exclusion criteria, forms the basis for this numbers-based analysis, which ensured our focus on high-quality, relevant studies.

**FIGURE 2:**
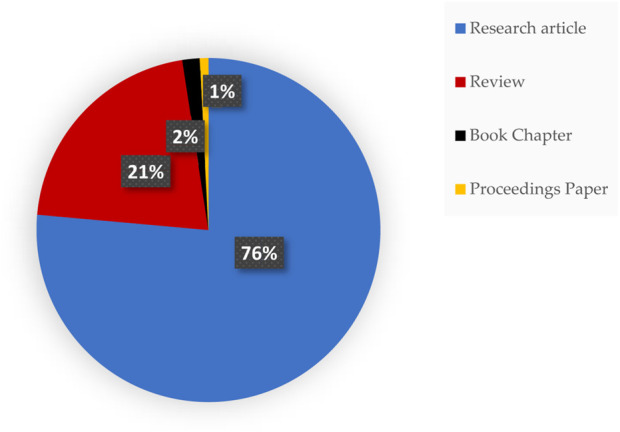
Proportion of publications by document type. Research articles comprise the majority of publications, followed by review articles, while book chapters and conference proceedings contribute minimally.

An analysis of publication types was conducted to assess the composition of the scholarly output within the retrieved literature. Figure 2 presents the distribution of publications by document type, showing that research articles constitute the majority of the literature (n = 94; 76.4%), followed by review articles (n = 26; 21.1%). Book chapters (n = 2; 1.6%) and conference proceedings (n = 1; 0.8%) contribute only marginally to the overall publication output.

### 3.2 Publication analysis based on journals

To understand the dissemination of research on telomere length in infectious diseases, we examined the journals that have contributed most actively to this field. Key outlets included *Frontiers in Immunology*, *PLoS ONE*, *Scientific Reports*, *AIDS*, *Cells*, and *Immunity & Ageing*. Table 1 summarises these journals’ total publications, citations, impact factors, and quartiles. For example, *PLoS ONE* published nine articles garnering 444 citations, while *Frontiers in Immunology* hosted six articles with 425 citations. The journals represented spanned the first (Q1) and second quarters (Q2).

**TABLE 1:** Summary of key journals publishing telomere-infection studies, including publication metrics, impact factors, and quartiles.

Rank	Source	Total publications	Total citations	Impact factor	Quartile
1	PLOS ONE	9	444	3.11	Q1
2	AIDS	6	340	3.10	Q2
3	Frontiers in immunology	6	425	5.90	Q1
4	Cells	4	180	5.20	Q1
5	Immunity & ageing	3	49	5.60	Q1
6	International journal of molecular sciences	3	38	4.90	Q1
7	JAIDS-journal of acquired Immune deficiency syndromes	3	72	2.20	Q2
8	Scientific reports	3	23	3.88	Q1
9	Ageing and disease	2	137	7.00	Q1
10	Aging-US	2	13	–	Q2

Key journals were identified by ranking sources in VOSviewer according to publication volume, with a minimum threshold of two publications. Citation metrics, impact factors, and journal quartiles were then considered to highlight the most influential outlets. Together, the top ten journals contributed 43 publications, representing about 35 percent of the total dataset, and underscoring their important role in disseminating research in this field.

**FIGURE 3:**
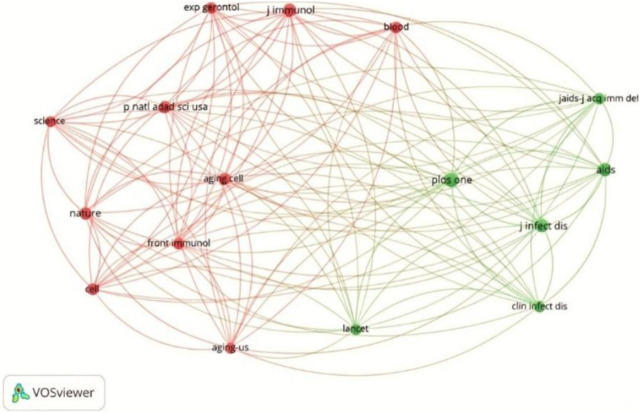
Network visualisation of co-cited journals publishing research on telomere length dynamics and infectious diseases. The map shows clusters of frequently co-cited journals (n = 16; minimum of 100 citations), indicating strong intellectual connections across immunology, infectious disease, and molecular biology.

To examine the intellectual structure and disciplinary foundations of research on telomere biology in infectious diseases, a journal co-citation analysis was performed. This approach identifies influential journals and reveals how knowledge is shared across related scientific domains. Figure 3 illustrates the resulting co-citation network, highlighting interconnected clusters that reflect cross-disciplinary linkages among immunology, infectious disease, and molecular biology.

### 3.3 Publication analysis based on authors

**FIGURE 4:**
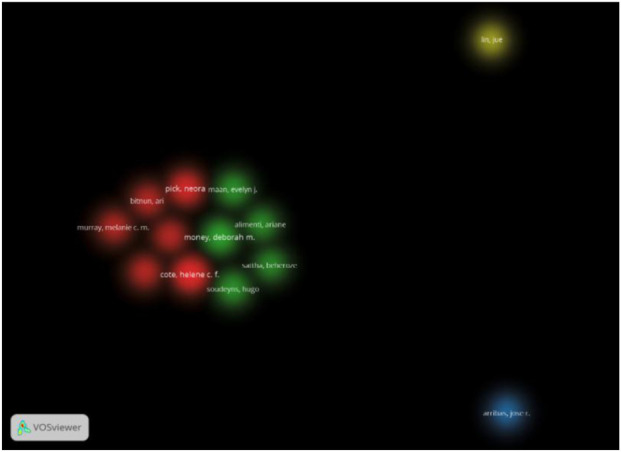
Co-authorship density map depicting collaborative networks among researchers in telomere biology and infectious diseases. Dense clusters are observed among Canadian researchers, with notable international collaborations, highlighting the research focus on HIV-related studies and the intensity of scholarly interactions within this field.

Analysis of publication contributions identifies several leading researchers. Côté HCF from Université de Montréal, Canada, contributed the highest number of publications (n = 9), followed by Pick N from the University of British Columbia, Canada (n = 8), and Maan EJ from British Columbia Women’s Hospital, Canada (n = 6). Key contributors include Money DM, Soudeyns H, Arribas JR, Bitnun A, Brophy J, Kakkar F, and Lin J, each with 4–5 publications (see Table 2 for detailed affiliations and counts). Canadian institutions dominate the research landscape, with strong collaborative ties evident among these authors. Figure 4, a co-authorship density map, visualises these networks, highlighting dense clusters of collaboration among Canadian researchers in HIV-related studies.

**TABLE 2:** Top 10 leading authors, Affiliations, and Publication Count.

Rank	Author	Publication	Affiliation	Country
1st	Cote HCF	9	Université de Montréal	Canada
2nd	Pick N	8	University of British Columbia	Canada
3rd	Maan EJ	6	Women’s health research institute, British Columbia Women’s hospital	Canada
4th	Money dm	5	British Columbia Women’s hospital, Vancouver	Canada
5th	Soudeyns H	5	Université de Montréal, Montreal	Canada
6th	Arribas JR	4	Institute for health research hospital La Paz (IdiPAZ), Madrid	Spain
7th	Bitnun A	4	University of Toronto, Toronto, Canada	Canada
8th	Brophy J	4	University of Ottawa, Ottawa, ON K1H 8L1	Canada
9th	Kakkar F	4	Centre hospitalier universitaire sainte-justine, université de Montréal, Montréal H3T 1C5, Québec	Canada
10th	Lin J	4	University of California San Francisco, San Francisco, California	US

To explore the collaborative structure within the field, co-authorship networks were analysed. Figure 4 visualises these networks, revealing concentrated clusters of collaboration among Canadian researchers, with strong links to international partners. The map highlights that research efforts are predominantly oriented toward HIV-related studies, reflecting both the intensity and specialization of scholarly activity in this domain.

### 3.4 Publication analysis based on countries/regions and institutions

The United States (29.3%), Canada (12.2%), and Spain (8.9%) topped publication outputs, with additional contributions from China, Italy, Brazil, France, Iran, Serbia, and Switzerland. Table 3 provides publication counts, single-country versus multi-country collaborations, and percentages. The United States led with 36 publications, including 10 international collaborations, while Canada contributed 15 nationally focused publications. Spain showed a high rate of multi-country collaboration (45.5%).

**TABLE 3:** Top 10 countries by publication metrics.

Rank	Country	Number of articles	Articles %	SCP	MCP	MCP %
1st	United States	36	29.3	26	10	27.8
2nd	Canada	15	12.2	13	2	13.3
3rd	Spain	11	8.9	6	5	45.5
4th	China	7	5.7	5	2	28.6
5th	Italy	6	4.9	4	2	33.3
6th	Brazil	5	4.1	5	0	0
7th	France	4	3.3	2	2	50
8th	Iran	4	3.3	3	1	25
9th	Serbia	4	3.3	4	0	0
10th	Switzerland	4	3.3	1	3	75

Key: USA, United States of America; SCP, single country publication; MCP, multiple country publication.

To visualise the global distribution of research output in telomere biology and infectious diseases, a geographical mapping analysis was conducted. This analysis highlights regional contributions and reveals disparities in scientific productivity across countries and institutions worldwide.

Global geographical distribution of scientific publications on telomere length dynamics and infectious diseases. Darker shades of blue indicate higher research output, with prominent contributions from the United States, Canada, Western Europe, China, and Australia.

**FIGURE 5:**
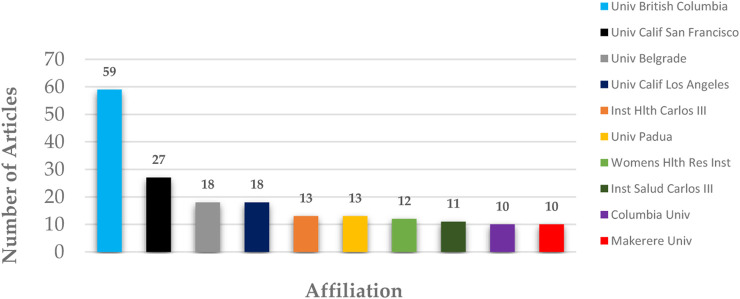
Distribution of publications across different institutions.

To identify the leading institutional contributors to research on telomere biology and infectious diseases, an institutional productivity and collaboration analysis was performed. This analysis highlights key research centres that play a central role in shaping the field, as shown in Figure 5.

Institutional distribution of scientific publications on telomere length dynamics and infectious diseases, highlighting major research centres, including the University of British Columbia, Université de Montréal, and the University of California, San Francisco.

### 3.5 Citation analysis

Citation analysis revealed key studies that have significantly shaped the research landscape on telomere biology and infectious diseases. The most highly cited work, The most highly cited work, Moro-García and Co (Moro-García et al., 2018) with 158 citations, explored the influence of inflammation on T lymphocyte differentiation. With 133 citations, which examined the relationship between aerobic fitness and immune ageing; and (Spielmann et al., 2011), another with 130 citations, which investigated the mechanisms of immunosenescence (Rodriguez et al., 2021). The top 10 cited works, their sources, and annual citation rates are summarised in Table 4.

**TABLE 4:** Top 10 highly cited articles related to immunosenescence, Telomere dynamics, and infection.

Rank	Author	Title	Source	Total citations	TC per year
1st	Moro-Garcia MA, 2018	Influence of inflammation in the process of T lymphocyte differentiation: Proliferative, metabolic, and oxidative changes	Frontiers in immunology	158	19.75
2nd	Spielmann G, 2011	Aerobic fitness is associated with lower proportions of senescent blood T-cells in man	Brain, behavior, and immunity	133	8.87
3rd	Rodriguez IJ, 2021	Immunosenescence study of T Cells: A systematic review	Frontiers in immunology	130	26.00
4th	Ilmonen P, 2008	Telomere attrition due to infection	Plos one	129	7.17
5th	Dock JN, 2011	Role of CD8 T Cell replicative senescence in human aging and in HIV-mediated immunosenescence	Aging and diseases	125	8.33
6th	Pathai S, 2013	Accelerated biological ageing in HIV-infected individuals in South Africa a case -control study	AIDS	119	9.15
7th	Cohen S, 2013	Association between Telomere length and experimentally induced upper respiratory viral infection in healthy adults	Journal of the american Medical association	110	8.46
8th	Jenny NS, 2012	Inflammation in aging: Cause, effect, or both?	Discovery medicine	109	7.79
9th	Effros RB, 2011	Telomere/telomerase dynamics within the human immune system: Effect of chronic infection and stress	Experimental gerontology	103	6.87
10th	Secher T, 2013	*Escherichia coli* producing colibactin triggers premature and Transmissible senescence in Mammalian cells	PLos one	96	7.38

Studies that are ranked by total citations in our dataset, represent foundational works shaping the field’s understanding of immunosenescence and telomere attrition.

### 3.6 Keyword and thematic analysis

To identify dominant research themes and emerging topics in the literature, a keyword-based thematic analysis was conducted using authors’ keywords. A word cloud visualization was generated to capture the most frequently occurring terms and to highlight prevailing areas of focus within the field.

**FIGURE 6:**
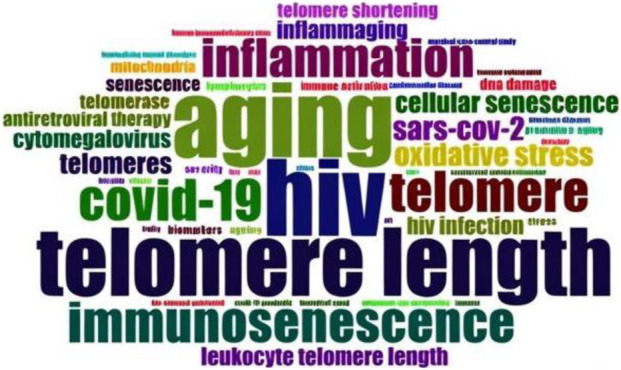
Word cloud of authors’ keywords with a maximum of 50 words. It illustrates the most frequent themes in research on telomere length dynamics and infectious diseases, with prominent emphasis on HIV, aging, telomere length, telomeres, and COVID-19.

A word cloud visualization (Figure 6) was generated from the authors’ keywords set to a maximum of 50 terms. Following a minimum occurrence threshold of five, 35 keywords met the inclusion criteria in VOSviewer. The nuanced themes captured included specific pathogens (e.g., hepatitis, tuberculosis) and molecular mechanisms (e.g., telomerase regulation) (35). The analysis revealed that HIV (26 occurrences), aging (23), telomere length (22), telomere (14), and COVID-19 (14) were the most frequently used keywords across publications. The dominant research focus has been on viral infections, immune aging, and telomere dynamics.

To examine the temporal evolution of research themes, an overlay visualization of keyword co-occurrence was generated using VOSviewer. This approach groups keywords according to their average year of publication, enabling the identification of emerging research hotspots and shifts in thematic emphasis over time. The colour gradient represents the average publication year, ranging from blue (earlier studies) to yellow (more recent studies), thereby illustrating the progression of research focus across distinct periods (Tomaszewski, 2023).

Although the bibliometric search strategy explicitly included bacterial, parasitic, and viral infections as primary search terms, analysis of the included publications revealed a strikingly uneven representation of infectious agents. As summarized in Table 5, viral pathogens dominated the literature, accounting for 110 of 123 studies (89.4%), with research almost exclusively focused on HIV-1, cytomegalovirus, hepatitis viruses, and SARS-CoV-2. In contrast, bacterial infections were represented by only 9 studies (7.3%), primarily involving *Mycobacterium tuberculosis* and selected periodontal pathogens, while parasitic infections were markedly underrepresented, with just 4 studies (3.3%) addressing *Plasmodium falciparum* malaria or parasitic coinfections.

**TABLE 5:** Distribution of infectious agents in the 123 publications included in this bibliometric review.

Pathogen category	Representative pathogens/diseases	Number of studies (n)	Percentage (%)	Key telomere-related mechanisms reported
Viruses	HIV-1, SARS-CoV-2 (COVID-19), hepatitis C virus (HCV), hepatitis B virus (HBV), cytomegalovirus (CMV), influenza, respiratory viruses	110	89.4	Chronic immune activation, telomerase dysregulation, oxidative stress, inflammation-driven replicative senescence, mitochondrial dysfunction
Bacteria	*Mycobacterium tuberculosis* (including multidrug-resistant TB), *Escherichia coli*, periodontal pathogens	9	7.3	Inflammation-induced telomere attrition, DNA damage responses, infection-related oxidative stress
Parasites	*Plasmodium falciparum* (malaria), parasitic coinfections	4	3.3	Acute inflammatory stress, oxidative damage, accelerated leukocyte telomere shortening
Total	—	123	100	—

## 4 Discussion

This bibliometric review elucidates the burgeoning intersection of telomere biology and infectious diseases, revealing how telomere dynamics serve as a molecular bridge between ageing, immune function, and pathogen interactions. Over the 2005–2025 period, publication trends demonstrate a robust upward trajectory, with an exponential increase during the COVID-19 era. This trend is likely driven by evidence linking shorter leukocyte telomeres to severe disease outcomes (Chebly et al., 2023; Aviv, 2020). Telomere research offers potential for infectious disease interventions, such as assessing vaccine efficacy in ageing populations with shortened telomeres or developing telomere-based diagnostics to predict infection severity (Heba et al., 2021). These applications could enhance personalised medicine approaches. This surge aligns with broader recognition of telomeres as biomarkers of cumulative infectious burden, where acute infections accelerate telomere shortening through rapid lymphocyte proliferation (Wakai et al., 2025a), and chronic conditions like HIV and tuberculosis exacerbate attrition via sustained inflammation and oxidative stress (Chebly et al., 2023; Macamo et al., 2024; Correia-Melo et al., 2014; Tunnicliffe et al., 2024).

Taken together, these bibliometric patterns reveals the clinical relevance of telomere biology in infectious diseases, positioning telomere length not only as a marker of immune aging but also as a potential predictor of disease severity, long-term immune dysfunction, and therapeutic or vaccine responsivenessy (Eisenberg et al., 2017). Emerging evidence suggests that shortened telomeres may also impair vaccine-induced immune responses by limiting lymphocyte proliferative capacity, particularly in older adults and individuals with chronic infections (Bulut et al., 2025). This highlights telomere dynamics as a potentially important, yet underexplored, factor in vaccine efficacy in infectious disease setting.

Journal analysis reveals the multidisciplinary nature of the research field. High-impact outlets, such as Frontiers in Immunology and PLoS ONE, have a dominant dissemination, with most works often in the Q1/Q2 quartiles, indicating rigorous peer review and broad accessibility. Co-citation networks (Figure 3) illustrate cross-pollination between immunology, molecular biology, and infectious disease journals, fostering integrative insights into mechanisms like telomerase regulation and shelterin complex stability (Mir et al., 2020; Vulliamy et al., 2011).

Contributions from authors and institutions reveal distinct geographic research hubs: Canadian researchers, notably Côté HCF and Pick N, have led advancements in HIV-related telomere studies, supported by robust co-authorship networks (Figure 4; Table 2). This prominence likely reflects strong funding frameworks and collaborative ecosystems, particularly at institutions like the University of British Columbia. In contrast, regions with high infectious disease burdens, such as Africa and Asia, remain underrepresented in the literature, despite the presence of relevant findings (Wakai et al., 2025a; Wang et al., 2019).

The dominance of the United States and Canada likely reflects robust research funding, advanced infrastructure, and established programs in telomere research. Conversely, underrepresentation of African countries may stem from limited research resources and focus on endemic diseases like malaria, highlighting global health disparities (USMAN, 2023; George et al., 2023; Rotimi et al., 2021).

**FIGURE 7:**
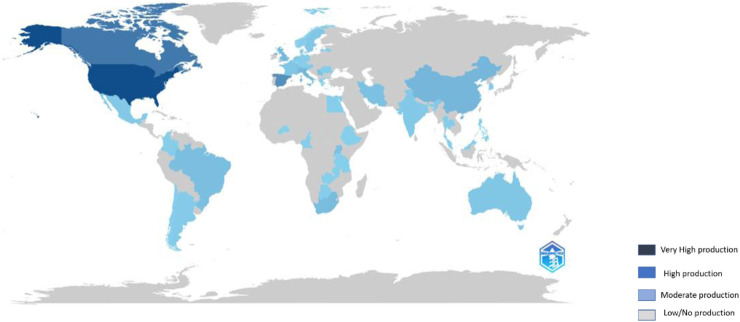
Geographical distribution of scientific production.

Country-level metrics (Table 3; Figure 7) reveal a Western-centric bias, with the United States leading in output and international collaborations, which may limit perspectives on endemic diseases such as malaria. However, a recent report by Wakai et al. (Wakai et al., 2025a) highlights that the impact of malaria on telomere length remains an understudied area despite increasing interest in telomere biology. Emerging studies have established a connection between malaria and telomere dynamics, underscoring the need for further investigation into this neglected field (Miglar et al., 2021; Miglar et al., 2020). Previous studies on malaria and telomere dynamics have primarily concentrated on the telomeres of the parasite itself (Bertschi, 2015; Calvo and Wasserman, 2016; Wakai et al., 2025b) or utilised animal models (Asghar et al., 2015; Asghar et al., 2016; Brown, 2022; Videvall et al., 2015), limiting insights into human telomere responses.

**FIGURE 8:**
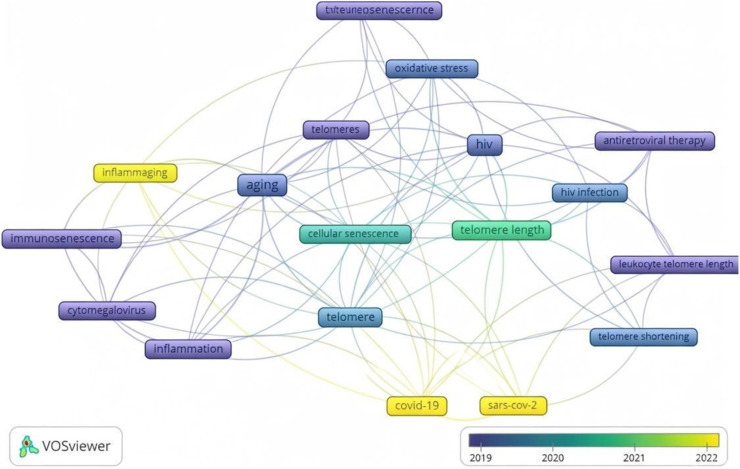
Overlay visualization of keyword co-occurrence network generated in VOSviewer, showing temporal progression of thematic clusters. It shows the temporal progression of thematic clusters in research on telomere length dynamics and infectious diseases. Earlier research (2005–2015) is predominantly associated with oxidative stress and HIV-related themes (blue), while more recent studies (2020–2025) emphasise COVID-19, host–pathogen interactions, and immune aging (yellow).

Citation analysis (Table 4) affirms the influence of seminal works on immunosenescence, such as (Moro-García et al., 2018). These studies provide mechanistic foundations for understanding how infections induce premature ageing, with implications for vulnerable populations (Heba et al., 2021; Noppert et al., 2020). The shifting themes in telomere–infectious disease research over the past 2 decades reflect the evolution of scientific interest alongside global health challenges. This change is evident in the patterns of keywords used across publications, which reveal where attention has grown and where knowledge gaps remain. Keyword analysis (Figure 8) reveals that earlier studies have primarily focused on oxidative stress, inflammation, and HIV-related telomere changes (Correia-Melo et al., 2014; Ahmed and Lingner, 2018). Infection-induced inflammation and oxidative stress—mediated in part by innate immune pathways, such as Toll-like receptor signalling—have been implicated in accelerated telomere shortening and immune cell senescence (Jose et al., 2017; Johnson et al., 2025). In recent years, attention has shifted toward COVID-19 and host–pathogen interactions, highlighting the increased interest in how pandemics influence immune ageing (Aviv, 2020; Tunnicliffe et al., 2024). The VOSviewer map visualises this shift clearly, pointing to emerging research areas and drawing attention to underexplored topics such as malaria (Tomaszewski, 2023). This trend highlights the crucial role telomeres play in immune regulation, as their shortening typically weakens immune responses and accelerates immune ageing (Heba et al., 2021; Bellon and Nicot, 2017). Yet, significant gaps remain. Addressing these gaps will deepen understanding of how infections affect telomere biology and long-term immune health.

The marked imbalance in pathogen representation is striking, especially in light of the substantial global burden posed by bacterial and parasitic diseases. This distribution highlights a strong bias toward viral pathogens with bacterial and especially parasitic infections underrepresented relative to their epidemiological impact. The shifting themes in telomere–infectious disease research over the past 2 decades reflect the evolution of scientific interest alongside global health challenges (George et al., 2023; Narita, 2023). This change is evident in the patterns of keywords used across publications, which reveal where attention has grown and where knowledge gaps remain. Keyword analysis (Figures 6, 8) reveals that earlier studies have primarily focused on oxidative stress, inflammation, and HIV-related telomere changes (Correia-Melo et al., 2014; Ahmed and Lingner, 2018). In recent years, attention has shifted toward COVID-19 and host–pathogen interactions, highlighting the increased interest in how pandemics influence immune ageing (Humaira et al., 2024; Anzaku and Afolabi, 2025). The VOSviewer map visualises this shift clearly, pointing to emerging research areas and drawing attention to underexplored topics such as malaria (Tomaszewski, 2023). This trend highlights the crucial role telomeres play in immune regulation, as their shortening typically weakens immune responses and accelerates immuneageing (Heba et al., 2021; Bellon and Nicot, 2017). Yet, significant gaps remain. Malaria continues to receive little attention despite evidence that it may influence telomere length, and bacterial infections are still far less studied than viral ones (Noppert et al., 2020). Addressing these gaps will deepen our understanding of how infections influence telomere biology and long-term imprints on immune system.

This disparity leaves a significant gap in telomere-focused research within the context of infectious diseases. Table 5 highlights this issue by clearly listing the pathogens that have been studied, while also drawing attention to those that remain largely overlooked despite their widespread endemic importance.

At the mechanistic level, the reported studies predominantly linked viral infections to telomere attrition through chronic immune activation, sustained inflammation, oxidative stress, and dysregulation of telomerase activity. In contrast, mechanistic insights related to bacterial and parasitic infections were sparse and largely indirect, reflecting the limited number of studies in these categories (Noppert et al., 2020). These findings indicate that current mechanistic understanding of telomere dynamics in infectious diseases is heavily shaped by viral models, with important implications for the generalizability of telomere-based interventions across diverse pathogen classes.

The particularly limited attention to malaria is noteworthy given its status as the most prevalent infection in lower-resource regions such as Africa and Southeast Asia. Despite evidence showing that malaria can significantly contribute to telomere shortening and accelerated cellular aging in populations from endemic areas (Band and Leffler, 2024; McQuillan et al., 2024), only a handful of studies have explored this relationship (Table 5). Wakai and Co recently reviewed works done relating malaria and telomere length (Wakai et al., 2025a), they reported the scarcity of data in this area which highlights a critical gap in our understanding of how malaria infection may influence telomere dynamics and emphasizes the need for focused research in these high-burden regions to better understand potential long-term health consequences.

The convergence of bibliometric trends and experimental findings highlights how research attention has clustered around specific mechanistic pathways linking infection and telomere attrition. Bibliometric trends, particularly the prominence of keywords related to oxidative stress, inflammation, and specific pathogens (e.g., HIV, tuberculosis, COVID-19; Figure 8, Table 5), directly reflect underlying biological mechanisms driving telomere attrition during infection. Infection-induced reactive oxygen species (ROS) accelerate telomere shortening via DNA damage response pathways, including p53 activation and shelterin complex disruption, leading to replicative senescence in immune cells (Correia-Melo et al., 2014; Ahmed and Lingner, 2018; Jose et al., 2017; Johnson et al., 2025). In chronic viral infections like HIV, sustained immune activation promotes repeated lymphocyte proliferation, exacerbating the end-replication problem and limiting telomerase activity in somatic cells (Adelakun et al., 2022; Macamo et al., 2024). Similar mechanisms operate in tuberculosis, where prolonged inflammation and ROS from mycobacterial persistence compound telomere erosion, contributing to immune exhaustion (Wang et al., 2019; Noppert et al., 2020).

Acute infections, such as COVID-19, trigger rapid clonal expansion of T cells, correlating with the post-2020 keyword surge and observed shorter telomeres in severe cases (Aviv, 2020). These trends cross-reference results showing HIV dominance (Figure 6) and recent COVID-19 emphasis, underscoring how chronic/persistent pathogens drive premature immunosenescence (Gray-Miceli et al., 2023). In contrast, underrepresented pathogens like malaria likely induce comparable stress through recurrent inflammation and hemolysis, yet human data remain sparse (Miglar et al., 2021; Wakai et al., 2025a; Miglar et al., 2020). Bacterial infections also lag, despite evidence of pathogen burden accelerating attrition (Noppert et al., 2020).

Limitations of this review include reliance on Web of Science data, which may underrepresent non-English or grey literature (Pranckutė, 2021; Guerrero-Bote et al., 2021) and exclusion of non-human studies, potentially overlooking translational insights. Web of Science data may underrepresent regional journals, particularly those from Africa and Asia, suggesting that future studies should cross-reference with Scopus or PubMed to capture a broader publication landscape (Guerrero-Bote et al., 2021). Future research should prioritise underrepresented pathogens, integrate multi-omics (e.g., genomics, proteomics) for causal inference, and explore telomere-targeted interventions, such as telomerase activators or anti-inflammatory agents, to mitigate infection-related ageing (Lee et al., 2025; Asghar et al., 2017). Longitudinal studies, such as those linking telomere length to infection outcomes, support telomeres as prognostic biomarkers for infectious disease severity and immune resilience (Noppert et al., 2020). The ethical implications of telomere research include privacy concerns in biomarker studies, as telomere length data could reveal health risks, necessitating robust data protection measures (Heba et al., 2021; Sanders and Newman, 2013). Future studies should move beyond descriptive bibliometric trends to directly integrate telomere measurements with clinical, immunological, and epidemiological data in diverse infectious disease settings, particularly in populations disproportionately affected by endemic infections.

## 5 Conclusion

This study provides a two-decade perspective on research exploring telomere biology in the context of infectious diseases. Herein, using bibliometric analysis, we show that while publications in this field have grown steadily, particularly in recent years during the COVID-19 pandemic, the research remains heavily skewed toward viral infections. Infections, caused by bacteria and parasites, especially malaria, which is caused by *P. falciparum*, despite their profound global impact on vulnerable populations, remain vastly under-reported. Evidence from a few sources suggests that malaria can accelerate telomere shortening and cellular ageing in populations living in endemic regions. The limited studies available leave an important research gap in understanding how high-burden infections shape telomere dynamics and leave long-term imprints. Looking ahead, there is a clear need for more research that focuses on infections affecting the world’s most vulnerable populations. Studying how diseases like malaria impact telomere biology could reveal important clues about the long-term effects of chronic and recurrent infections on the immune system. This review aims to encourage future studies that incorporate multidisciplinary approaches-such as molecular, clinical, and epidemiological perspectives-and foster collaborations across disciplines and regions, thereby helping to develop the field of telomere biology research that is both more comprehensive and directly relevant to health challenges plaguing our communities.
